# A method for estimating coherence of molecular mechanisms in major human disease and traits

**DOI:** 10.1186/s12859-020-03821-x

**Published:** 2020-10-21

**Authors:** Mikhail G. Dozmorov, Kellen G. Cresswell, Silviu-Alin Bacanu, Carl Craver, Mark Reimers, Kenneth S. Kendler

**Affiliations:** 1 Department of Biostatistics, Virginia Commonwealth University, Richmond, VA USA; 2 Department of Pathology, Virginia Commonwealth University, Richmond, VA USA; 3 Virginia Institute for Psychiatric and Behavior Genetics and the Department of Psychiatry, Virginia Commonwealth University, Richmond, VA USA; 4 Department Physiology, Michigan State University, East Lansing, MI USA; 5 Department Biomedical Engineering, Michigan State University, East Lansing, MI USA; 6 Philosophy-Neuroscience-Psychology Program, Washington University in St. Louis, St. Louis, MO USA

**Keywords:** GWAS, Network, Degree, Coherence

## Abstract

**Background:**

Phenotypes such as height and intelligence, are thought to be a product of the collective effects of multiple phenotype-associated genes and interactions among their protein products. High/low degree of interactions is suggestive of coherent/random molecular mechanisms, respectively. Comparing the degree of interactions may help to better understand the coherence of phenotype-specific molecular mechanisms and the potential for therapeutic intervention. However, direct comparison of the degree of interactions is difficult due to different sizes and configurations of phenotype-associated gene networks.

**Methods:**

We introduce a metric for measuring coherence of molecular-interaction networks as a slope of internal versus external distributions of the degree of interactions. The internal degree distribution is defined by interaction counts within a phenotype-specific gene network, while the external degree distribution counts interactions with other genes in the whole protein–protein interaction (PPI) network. We present a novel method for normalizing the coherence estimates, making them directly comparable.

**Results:**

Using STRING and BioGrid PPI databases, we compared the coherence of 116 phenotype-associated gene sets from GWAScatalog against size-matched KEGG pathways (the reference for high coherence) and random networks (the lower limit of coherence). We observed a range of coherence estimates for each category of phenotypes. Metabolic traits and diseases were the most coherent, while psychiatric disorders and intelligence-related traits were the least coherent. We demonstrate that coherence and modularity measures capture distinct network properties.

**Conclusions:**

We present a general-purpose method for estimating and comparing the coherence of molecular-interaction gene networks that accounts for the network size and shape differences. Our results highlight gaps in our current knowledge of genetics and molecular mechanisms of complex phenotypes and suggest priorities for future GWASs.

## Background

Genome-wide association studies (GWAS) have significantly advanced our understanding of complex phenotypes by identifying disease- and trait-associated genetic markers and suggesting corresponding genes [1, 2]. However, GWAS findings explain only a fraction of heritability [3–5]. Furthermore, the corresponding genes are often spread across different chromosomes with no known connection to one another, hindering understanding of the molecular mechanisms. These limitations of current generation GWAS might stem from the yet incomplete knowledge of genetic determinants of complex phenotypes, the potential heterogeneity and/or the causes other than genetics [6–8].

Many studies have shown that genetically-driven phenotypes are often associated with functionally related genes that are more likely to form networks of interacting protein products [9–15]. This observation has been verified systematically for a large number of diseases [16], thus confirming a fundamental hypothesis of the interactome-based approach to understanding human phenotypes, namely that disease- and trait-associated genes tend to form interaction modules [17]. Such interaction modules may be viewed as connected subnetworks within the full interactome and may contain all the molecular determinants of a certain phenotype. Consequently, methods to quantitatively assess these phenotype-associated networks of functionally-related genes have been developed [10, 13, 18].

Network properties, such as connectivity (aka degree distribution) [15, 19–21], can be compared to better understand the relationships among phenotypes [22–24]. Networks formed by interactions among phenotype-associated genes, or, more precisely, their protein products, are thought to have high connectivity [10, 11, 17]. This intuition reflects a well-known “guilt-by-association” principle that genes (or, more precisely, their products) forming an interaction network are more likely to have similar functions [25] and co-expression patterns [11, 26]. Although this view has been criticized [27], several studies consistently observed phenotype-associated genes to be either connected as a single network or to participate in common phenotype-specific subnetworks [16, 18, 28–30]. Therefore, high connectivity among phenotype-specific genes may indicate coherent molecular mechanisms that could be targeted therapeutically. In terms of graph theory, we are asking whether a phenotype-specific network is a community—a highly connected subgraph relatively well-separated from the rest of the network [31]. However, the direct comparison of connectivity across phenotype-specific networks is hindered by the fact that different phenotypes are associated with different numbers of genes forming networks of different sizes and configurations.

This study presents a novel measure of network coherence as a slope of the internal versus external degree distributions. The internal degree distribution is defined as gene-specific interaction counts within a protein–protein interaction (PPI) network formed by phenotype-associated genes, while the external degree distribution counts interactions with other genes in the whole PPI network. We used selected gene sets from the MSigDb database [32] as a reference for highly coherent networks, while sets of randomly sampled genes were used as the reference for the absence of coherence. Using MSigDb and random networks matched in size to phenotype-specific networks, we derived a normalized measure of coherence that can be compared across phenotypes. We hypothesized that the level of coherence may inform us about similarities and differences among phenotypes. Using two PPI databases (STRING [33] and BioGrid [34]), we compared coherence of 133 phenotypes from GWAScatalog [35, 36]. Our results show the tendency of intelligence-related traits to have low coherence and metabolic traits and diseases to have high coherence. Our method enables direct comparison of coherence measures and highlights gaps in the current understanding of molecular mechanisms of many phenotypes, e.g., Major Depressive Disorder having the lowest coherence in the already low-coherence “Psychiatric disease” category.

## Results

### Phenotype-specific networks of protein–protein interactions (PPIs)

We collected 133 phenotype-associated gene lists from the NHGRI-EBI GWAS catalog [36] (see Methods). They were grouped into seven disease categories (“Autoimmune”, “Cancer”, “Cardiovascular disease”, “Eye disease”, “Metabolic disease”, “Neurologic”, “Psychiatric”) and five trait categories (“Anthropometric trait”, “Cardiovascular trait”, “Eye trait”, “Intelligence”, “Metabolic trait”, Table 1, Additional file 1).

**Table 1:** Summary statistics of the analyzed categories

Category	Number of SNPs	Number of genes	Total diseases	Mean biogrid coherence	Mean string coherence	Mean string filtered coherence
**Trait**						
Eye trait	158	109	5	NA	0.724	1.050
Metabolic trait	790	646	10	0.738	0.779	0.863
Anthropometric trait	2090	1771	15	0.460	0.637	0.691
Cardiovascular trait	728	604	15	0.555	0.675	0.625
Intelligence	181	177	4	0.180	0.395	0.405
**Disease**						
Metabolic disease	331	269	4	0.470	0.825	0.907
Cancer	670	570	15	0.490	0.689	0.723
Neurologic	372	333	7	NA	0.493	0.718
Autoimmune	1383	1184	18	0.425	0.820	0.701
Eye disease	229	202	5	0.330	0.630	0.663
Cardiovascular disease	301	259	7	0.380	0.694	0.660
Psychiatric	678	611	11	0.540	0.552	0.642

Average coherence estimates are shown. “NA” values indicate that a category lacked phenotypes with a sufficient number of PPIs. Sorted by “String Filtered” average coherence

We created networks of phenotype-associated genes using PPI information from STRING [33] and BioGrid [34] databases (see Methods). We used three types of PPI data. First, STRING data filtered by interaction confidence score above 500 (the middle of the bimodal distribution of the score, 0–1000 range, referred hereafter as “STRING filtered”) was used as the primary source of curated PPI data. This filtering step selects for only high-confidence PPIs. The advantage of using filtered data is the reliance on high-quality PPIs. The disadvantage is that, for some phenotypes, the number of PPIs may be insufficient for forming networks. Consequently, these phenotypes were removed from the analysis (see Methods) leaving 116 phenotype-associated gene sets that could be analyzed. Second, full STRING data (henceforth, “STRING”) was used to maximize the use of PPI information at the expense of potential noise. The advantage of using complete data is that more trait-associated genes will have PPI information and, hence, can be analyzed. The disadvantage, however, is that the results may be less reliable due to the presence of noisy PPIs with low confidence scores. Third, BioGrid PPI data, which has been curated to keep only high-confidence interactions, was used. The use of different data sources (STRING and BioGrid) and filtering (full or filtered STRING data) was intended to increase the generalizability of our conclusions.

### Degree distribution as a measure of coherence

A typical network consists of nodes connected by edges [21]. In genomics, nodes typically represent genes and edges correspond to some measure of interactions, e.g., gene co-expression or interactions between protein products [37, 38]. In terms of phenotypes, an intuitive expectation is that they will be represented by coherent networks of functionally related genes, similar gene expression profiles, shared genomic variants, higher PPI interactions, and higher co-morbidity [16, 17, 38–42], reviewed in [24, 43]. In terms of graph theory, a coherent network is a community consisting of a group of nodes that are relatively highly connected to each other but sparsely connected to other nodes in the global network [31, 44].

Numerous network properties have been defined to describe network structures [23, 38]. A degree of a node, or connectivity, is one of the most fundamental characteristics defining the number of other nodes connected to a given node. A collection of degrees of all network nodes forms a degree distribution. Comparing degree distributions among networks is an intuitive way to gain an understanding of network similarities and differences in terms of connectivity (aka coherence) of the corresponding genes [15, 21]. We hypothesized that comparing degree distributions of networks formed by phenotype-associated gene sets may inform us about similarities and differences among phenotypes in terms of coherence estimates, informing us about the underlying molecular mechanisms.

### Internal versus external degree distributions as a measure of coherence

Given that phenotype-specific networks occur within the global network of PPIs, we developed a metric of coherence based on the level of gene interactions. This metric is inspired by previous work showing that communities within a large network have high internal but low external levels of interactions (degrees) [45, 46]. Thus, for each phenotype-associated PPI network, we considered the relationship between its internal and external degree distributions. The internal degree distribution is defined by considering gene-specific edges within an isolated network formed by phenotype-associated genes. The external degree distribution is defined by considering gene-specific edges to other genes in the whole PPI network. Intuitively, a highly coherent network is expected to have a large number of internal edges but a relatively small number of external edges. An extreme example of high coherence would be a fully internally connected network with zero interactions with external genes. Conversely, a low-coherence network is expected to have a relatively low number of internal edges similar to the number of external edges.

To compare coherences, we estimated coherence as the slope of a line through a scatterplot of internal versus external degree distributions. That is, we visualized internal (X-axis) versus external (Y-axis) degrees for each phenotype-specific gene on a single plot and fit a regression line through it, enforcing the fit through the origin. A network with the highest coherence (full internal connectivity, zero external connections) would be represented by a horizontal line. Conversely, a network with the lowest coherence (zero internal connectivity, full external connectivity) would be represented by a vertical line. Consequently, slopes of internal versus external degree distributions of phenotype-specific networks would represent the corresponding levels of coherence. In summary, comparing internal versus external degree distributions represents a viable metric to quantify and compare the molecular coherence of phenotype-associated networks.

### Networks of KEGG/REACTOME pathways and GO Cellular component collection versus randomly selected genes serve as references for high versus low coherence, respectively

The coherence of phenotype-associated gene sets should be measured with respect to the realistic references of high and low coherence. To establish a reference of high coherence, we considered networks from collections of the Molecular Signatures Database (MSigDB v6.2) [32]. MSigDB contains sets of genes having various types of functional relationships. We found that pathways from the Kyoto Encyclopedia of Genes and Genomes (KEGG) [47] had the smallest average slope corresponding to the highest level of coherence (Additional file 2). Networks assembled from Gene Ontology-Cellular Component (GOCC) collection and REACTOME pathways followed, representing alternative sources of networks with high coherence. This is expected as genes expressed in the same cellular components (GOCC genes) or participating in the same metabolic pathways (REACTOME genes) are presumed to interact more frequently. In our study, KEGG networks were used in parallel with GOCC and REACTOME (referred hereafter as MSigDb networks) as a reference for high coherence. Conversely, as a reference for low coherence, we randomly sampled genes from a pool of genes in a given PPI database. These random gene lists represent expected network coherence arising by chance. The slopes of MSigDb and random networks represent reference levels for high and low coherence, respectively.

### Network size and configuration hinders direct comparison of degree distributions

**Fig. 1:**
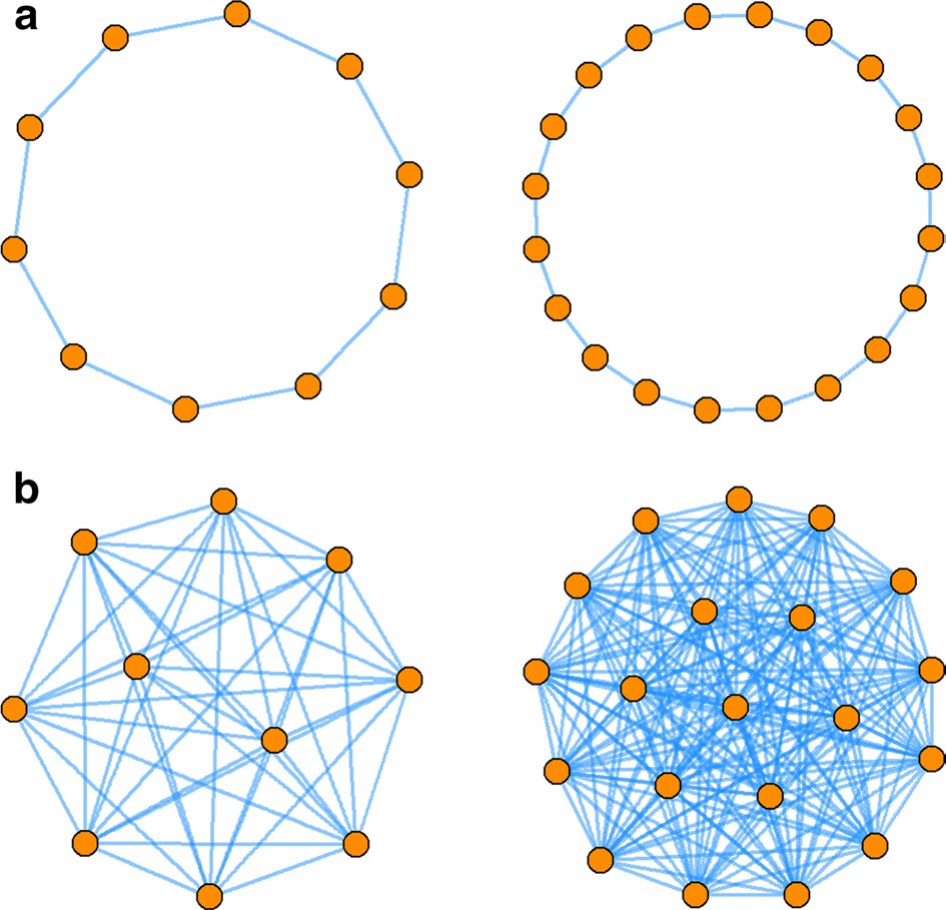
Degree distributions are affected by network size and configuration. Degree distributions of **a** ring networks and **b** fully connected networks containing 10 and 20 nodes. While degree distributions of similarly coherent ring networks can be directly compared, degree distributions of fully connected networks depend on network size

Direct comparison of degree distributions of the networks formed by phenotype-specific genes is hindered by the fact they depend on network size and configuration [48]. An intuitive example is to consider a ring and a fully connected network of size 10 and 20 nodes, respectively (Fig. 1). The degree distributions of ring networks can be directly compared regardless of network size (vectors of “2” of length 10 and 20, respectively, Fig. 1a). Although the fully connected networks are similar in that they are fully connected (i.e., similarly coherent), their degree distributions differ (all nodes in the 10-node network have a degree “9”, while all nodes in the 20-node network have degree “19”, Fig. 1b). These observations highlight the difficulty in comparing degree distributions of phenotype-associated gene networks due to two facts: (1) the network sizes differ, and (2) the network configuration (e.g., fully connected, ring, or intermediate connectivity) is unknown.

**Fig. 2:**
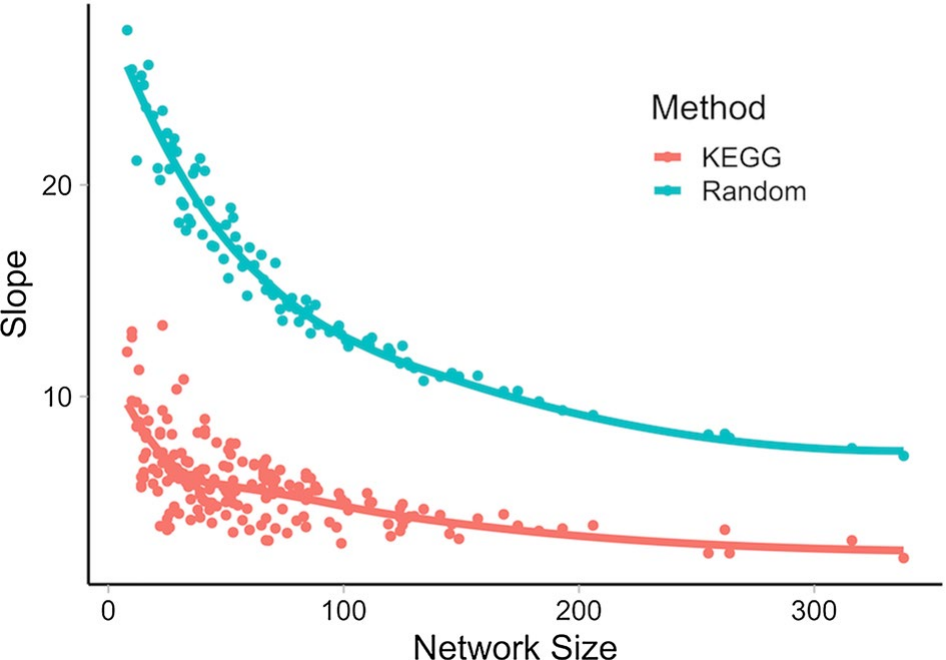
Network size is inversely associated with coherence. Slopes of internal versus external degree distributions (aka coherence) for KEGG and random networks are plotted against network size

To observe whether network properties affect coherence estimation in experimental settings, we investigated the effect of network size on the slopes of KEGG and random networks. We observed substantial negative correlation between network sizes and the slopes of KEGG and random networks (Pearson Correlation Coefficient (PCC) − 0.58/− 0.86, respectively, Fig. 2, Additional file 2). Similarly, slopes of the phenotype-specific networks and sizes of the corresponding confidence intervals negatively correlated with the number of genes in the networks (Average PCC − 0.49/− 0.70, respectively, Additional files 3, 4). This is expected as network size affects internal degree distributions and the corresponding slopes on the internal versus external degree plots, with the larger networks allowing for a more precise estimation of slopes (smaller confidence intervals). These observations confirm the notion that coherence estimation using internal versus external degree distributions should be controlled for network size.

### Network size-independent estimation of coherence

Given the dependence of degree distributions on network size, we designed a simple strategy to account for it. Briefly, for each phenotype-specific network of a given size, we created size-matched references of high and low coherence. Specifically, we selected 30 MSigDb networks (10 from KEGG, GOCC, and REACTOME collections) and 100 random networks matched in size to the corresponding network of phenotype-specific genes. Consequently, we use median slopes of internal versus external degree distributions created by these size-matched references to normalize the slopes of phenotype-specific networks to the [0, 1] range (see Methods), where 0/1 correspond to random/high coherence, respectively. We found that the use of size-matched references indeed alleviates the dependency of coherence on network size (Average PCC = 0.01, Additional file 4) and allows for direct comparison of normalized coherences across phenotypes. A schematic overview of our method is shown in Additional file 5.

### Results derived from different PPI data are largely consistent

In addition to size dependency, coherence estimates depend on the choice of the PPI database used to build networks and estimate the corresponding internal versus external degree distributions. To evaluate the consistency of coherence estimates, we assessed the pairwise correlation of normalized coherences obtained using different PPI databases. Correlation between coherence estimates obtained using “STRING” and “STRING filtered” was high (PCC = 0.80, Additional file 4). Expectedly, coherence estimates using Biogrid database were less similar to that of “STRING” and “STRING filtered”; however, the overall correlation remained high. Notably, coherence estimates using “STRING filtered” database were most similar to that of “Biogrid” (PCC = 0.58), indicating that filtering step indeed removes “noisy” PPIs and improves quality of coherence estimates. Overall, these results demonstrate consistency in coherence estimates using different PPI databases.

### Networks with high/low coherence have distinct degree distributions

**Fig. 3:**
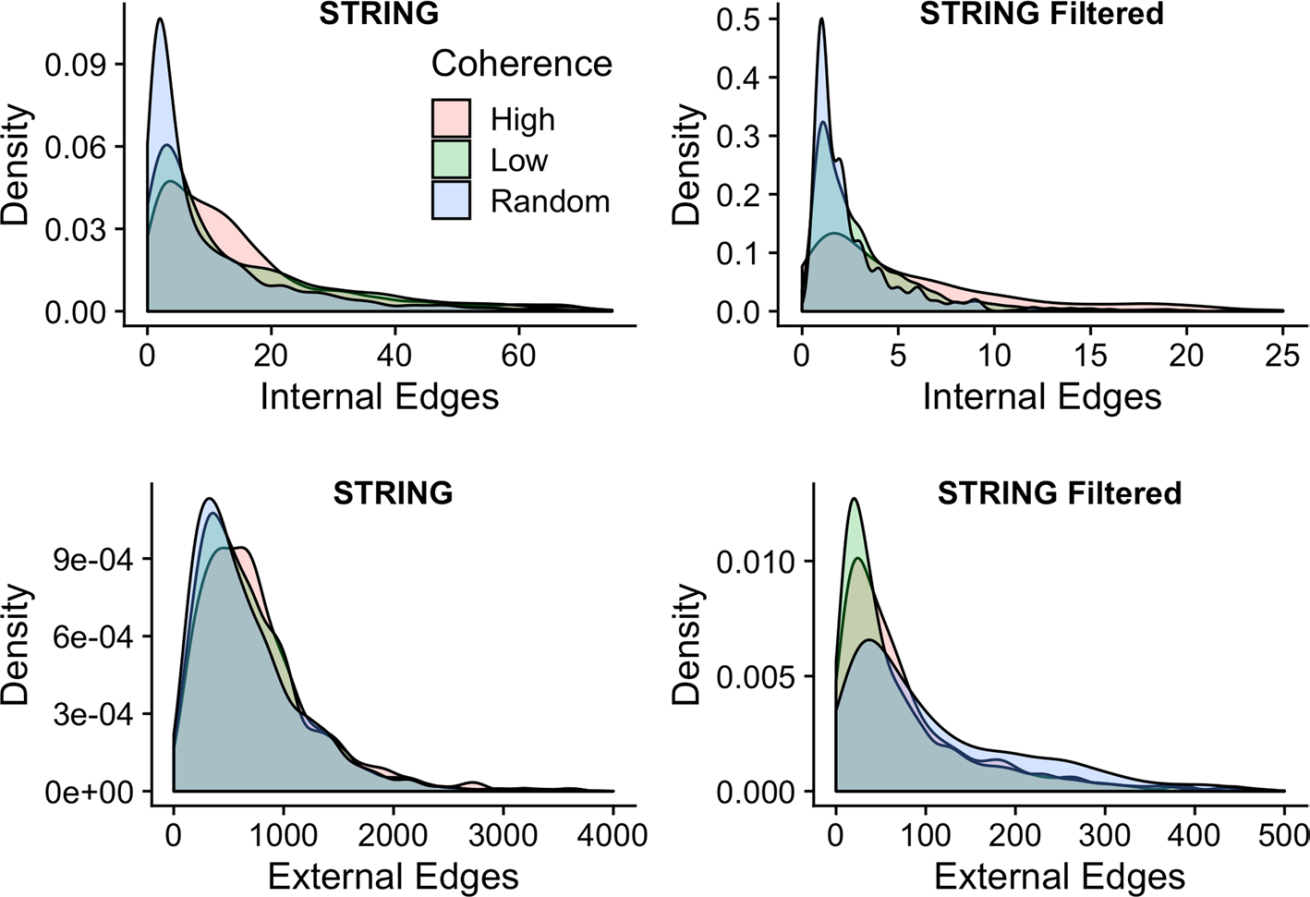
Degree distributions differ for high/low-coherence networks. Internal and external degree distributions for high- and low-coherence networks and size-matched random networks. STRING and STRING filtered indicate the corresponding PPI database. Bonferroni-corrected Wilcoxon *p* values comparing the distributions are in Additional file 10

Our measure of network coherence is expected to be proportional to the level of internal and/or external degree distributions. To investigate the relationship between coherence and degree distributions, we selected phenotype networks with low and high coherence. The thresholds for low and high normalized coherence were selected as < 0.58 and > 0.85, corresponding to the first and third quartiles of the coherence range, respectively. The Biogrid-generated networks were omitted because they had insufficient number of genes to form well-defined degree distributions. Expectedly, the internal degree distributions for high- and low-coherence networks were significantly different from random (Bonferroni-corrected two-tailed Wilcoxon *p* values < 5.30E−12), and these results were consistent when using different PPI databases (Fig. 3, Additional file 10). Similarly, the internal degree distribution for high-coherence networks was significantly larger than that of low-coherence networks (*p* value = 2.01E−16/2.12E−4 for STRING/STRING filtered databases, respectively). Similar observations were observed for external degree distributions of high- and low-coherence networks (Fig. 3, Additional file 10). In summary, these results support the notion that networks of high coherence have significantly more internal and external interactions as compared with low-coherence and random networks.

### Metabolic and intelligence-related traits as examples of networks with overall highest/lowest coherence

**Fig. 4:**
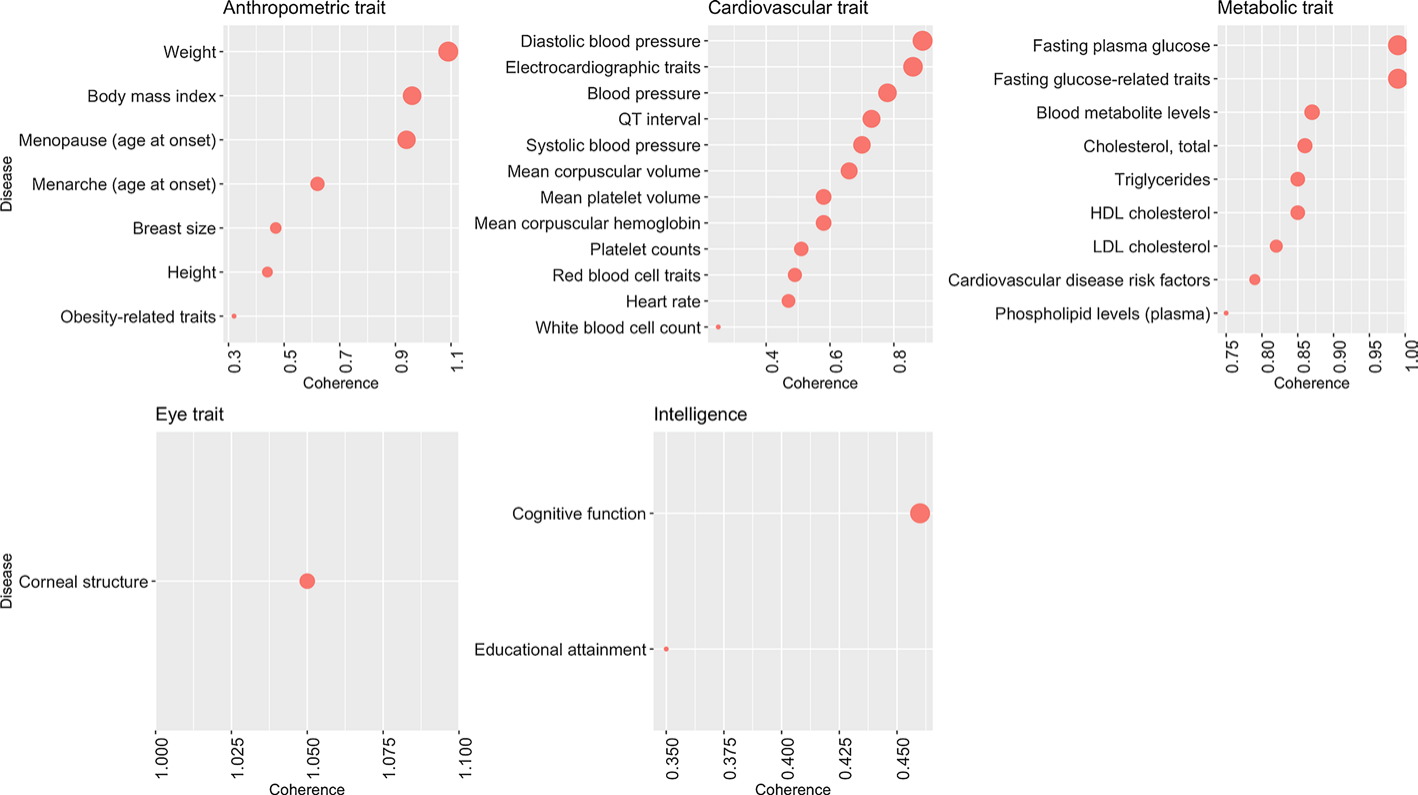
Coherence estimates of traits. Size of dots represents the level of normalized coherence (X-axis) for individual traits (Y-axis)

We estimated coherence of 49 traits from five categories (Fig. 4, Additional files 3, 6, 7). Among anthropometric traits, Weight and Body Mass Index had the highest level of coherence. Other examples of traits with high coherence include blood pressure-related traits (cardiovascular) and glucose-related traits (metabolic). Notably, the metabolic trait category had the highest overall coherence (mean coherence 0.86, Table 1), and these results were consistent when using other PPI databases (Additional file 1). Several notable examples of low coherence include Obesity-related traits, Height (anthropometric traits), and blood count-related traits (cardiovascular). Traits in the Intelligence category had low overall coherence (mean coherence = 0.41).

### Metabolic diseases and cancer networks have high coherence

**Fig. 5:**
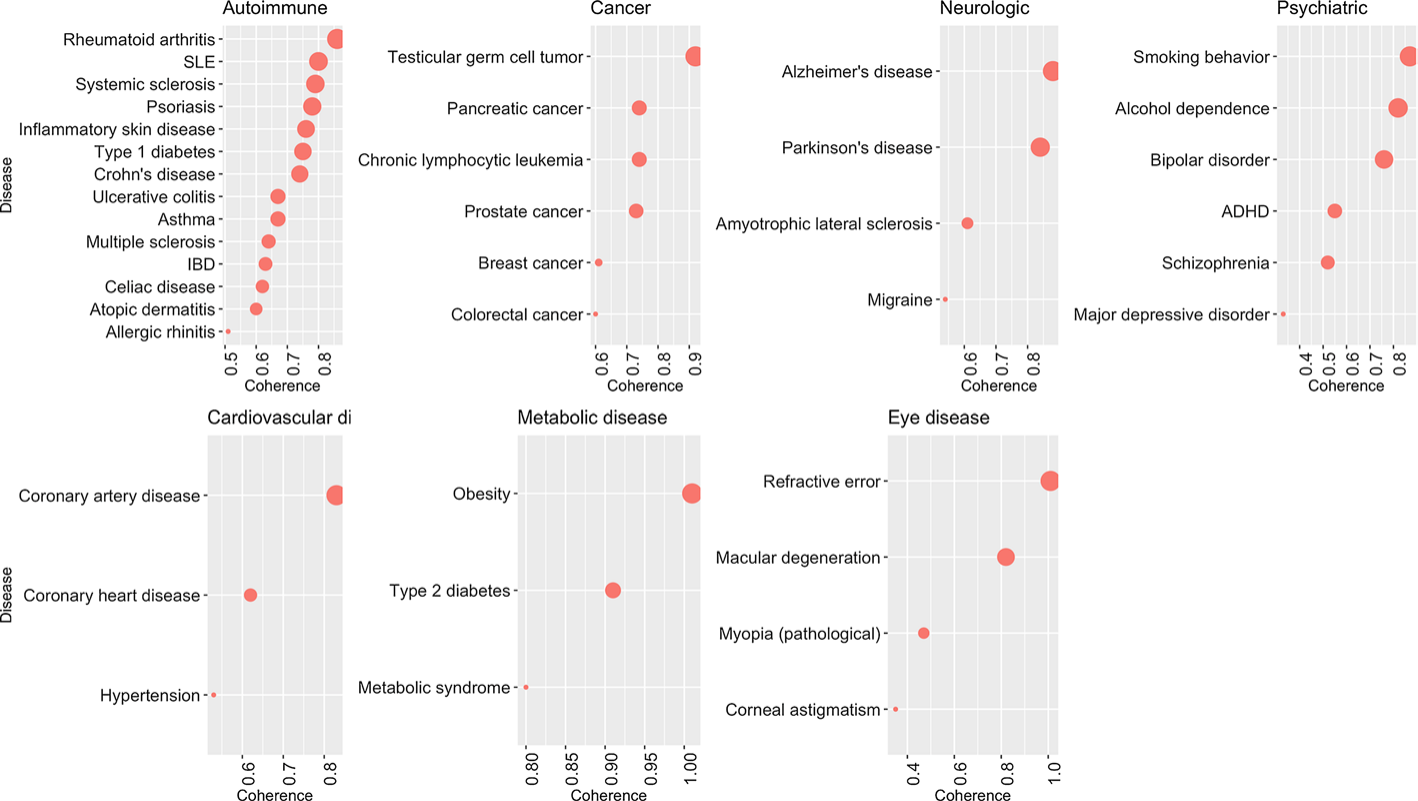
Coherence estimates of diseases. Size of dots represents the level of normalized coherence (X-axis) for individual diseases (Y-axis)

We further estimated coherence of 67 diseases from seven categories (Fig. 5, Additional files 3, 8, 9). Metabolic diseases had high overall coherence (average coherence 0.91, Table 1), with “Obesity” and “Type 2 diabetes” being the most coherent (1.01 and 0.91, respectively). Similarly, the average coherence for cancer diseases was 0.72, with “Testicular germ cell tumor” and “Pancreatic cancer” being highly coherent (0.92 and 0.74, respectively).

### Intermediate coherence of autoimmune, neurologic, and eye diseases

“Rheumatoid arthritis”, “Systemic Lupus Erythematosus”, and “Systemic sclerosis” were the most coherent in the “Autoimmune” category (0.86, 0.80, 0.79, respectively). On the other end of the coherence spectrum, “Allergic rinitis” and “Atopic dermatitis” were the least coherent (0.51, 0.60, respectively). The average coherence of autoimmune diseases was high, 0.70 (Table 1), with all autoimmune diseases, except “Allergic rinitis”, having coherence estimates > 0.60 (Additional file 3). Diseases in the “Neurologic” category were similarly highly coherent (average coherence 0.72). However, the average coherence of neurologic disorders using STRING database was 0.49, suggesting that, with larger number of networks being estimated the overall coherence of neurologic diseases is low. “Alzheimer’s disease” and “Parkinson’s disease” were the most coherent (0.88 and 0.84, respectively), while “Migraine” was the least coherent (0.54). Diseases in the “Eye disease” category were more heterogeneous, with coherence ranging from 0.35 for “Corneal astigmatism” to 1.01 for “Refractive error”. The average coherence of diseases in the “Eye disease” category was relatively high (0.66).

### Psychiatric and cardiovascular diseases as examples of low coherence

Diseases in “Psychiatric” and “Cardiovascular” categories showed the lowest average level of coherence (0.64 and 0.66, respectively, Table 1). The corresponding coherence estimates were highly heterogeneous. Among cardiovascular diseases, “Coronary artery disease” showed the highest level of coherence (0.83), while “Hypertension” had the lowest coherence (0.53). Among psychiatric disorders, “Smoking behavior” and “Alcohol dependence” had high coherence (0.87 and 0.82, respectively). Notably, coherences of “Schizophrenia” and “Major depressive disorder” networks (0.52 and 0.33, respectively) were at the lower end of coherence estimates among all phenotypes. Other psychiatric disorders, such as “Bipolar disorder”, “ADHD”, showed intermediate coherence estimates (0.76 and 0.55, respectively).

### Coherence of most phenotypes is significantly larger than random networks

Given the wide range of phenotype-specific coherence estimates, a natural question is whether they are significantly larger than the coherence of random networks. We assessed the significance of coherence estimates using a permutation test (see Methods), individually for each PPI database. Permutation p-values obtained using Biogrid and STRING filtered databases were highly correlated (PCC = 0.64, Additional file 4), as would be expected for high-quality PPIs. Permutation p-values using STRING databases were predominantly significant (*p* value < 0.001) due to a large number of PPIs that enabled phenotype-specific networks to be consistently different from random. Permutation p-values inversely correlated with coherence estimates (average PCC = − 0.23), as would be expected for less coherent networks that resemble random networks. This dependency was especially pronounced for database-specific estimates, e.g., Biogrid coherence and p-value estimates correlated with PCC = − 0.69 (STRING filtered PCC = − 0.53, Additional file 4). These observations confirmed that coherence and p-value estimates remained consistent when using different PPI databases.

The majority of phenotype-specific networks (~ 85%) were significantly more coherent than random networks (STRING filtered *p* value < 0.001, Additional file 3). Among less significant phenotypes were “White blood cell count” (*p* value = 0.085) and other blood count-related phenotypes, “Corneal astigmatism” (*p* value = 0.04), “Major depressive disorder” (*p* value = 0.03). When using Biogrid databases, “Educational attainment”, “Myopia (pathological)”, and “Celiac disease” were among the non-significant phenotypes (*p* values 0.30, 0.20, 0.16, respectively). Interestingly, “Schizophrenia” network, despite being among the least coherent, was consistently significantly different from random (*p* value < 0.0001). Overall, the significance estimation of coherence supports our observation that phenotype-specific networks in “Intelligence” and “Psychiatric disease” categories generally have low coherence that is frequently statistically insignificant. In summary, these results confirm our ability to identify coherence of phenotype-specific networks and test their significance over random networks.

### Coherence captures distinct network properties than modularity

Our study considers a network of phenotype-associated genes in its entirety. However, networks may be comprised of interacting modules, i.e., relatively isolated subnetworks [9]. Such subnetworks, while highly coherent by themselves, would have relatively low coherence overall due to the limited number of interactions among them (canonical definition of subnetwork). Consequently, a network with low coherence may consist of several modules and have high modularity. In turn, a network with high coherence may be represented by a single module and, consequently, have low modularity. Our assessment of network modularity using STRING and STRING filtered PPIs revealed a negative association of modularity with network size, that is, the larger the network is, the less modular it becomes (Additional files 3, 4). We also did not observe a measurable association between modularity and coherence. Notably, network modularity using BioGrid PPIs showed a moderate positive association with network size and coherence, implying that, as networks get larger, they become more modular and yet highly coherent. Investigation of individual phenotype-specific networks built using BioGrid PPIs revealed they are expectedly smaller than networks build using STRING PPIs, making modularity estimates imprecise. In summary, our results did not reveal a consistent association between coherence and modularity, suggesting these metrics capture distinct network properties.

Although our results did not show an association between coherence and modularity, our study is limited by using a single most common modularity measure [49]. Although numerous methods for module detection have been described [42, 50–52], comparing modules between diseases is plagued by the size-dependend normalization issues described in the Results section. Our future direction includes the development of a strategy for normalization of modularity by network size and comparing it with normalized coherence.

## Discussion

We developed a general-purpose method to measure and compare the coherence estimates of the molecular mechanisms in networks of genes. Intuitively, gene networks with high internal and low external number of interactions (degrees) would be considered highly coherent; consequently, coherence is defined as the slope through the internal and external degree distribution scatterplot. We normalized coherences (slopes) to the range of high and random coherence, exemplified by MSigDb and random networks. To decouple coherence from its dependence on network size, we used MSigDb and random networks matched in size to phenotype-specific networks. Applied to the analysis of 116 disease- and trait-associated gene sets, we found that metabolic diseases and traits had overall high coherence, while psychiatric and cardiovascular diseases generally had lower coherence. These results were consistent when using different PPI databases. Our method allows one to quantify and compare the estimated coherence of molecular mechanisms across phenotypes, and will only improve as more PPI information becomes available.

The coherence measure allows us to gain insights into the genetic component of various phenotypes, as revealed by the current state of the corresponding GWASs. The high coherence of metabolic diseases and traits arguably reflects the strong genetic component driving molecular mechanisms of metabolism-specific gene networks. On the contrary, the low coherence of a phenotype suggests either insufficient knowledge of genetics (as represented in current-generation databases) or the true lack of such a strong genetic influence, (and correspondingly, the heightened importance in these cases of non-genetic, and possibly environmental, factors. If the incoherence results from insufficient knowledge, this should be remedied as novel genes are identified through larger-scale GWASs and meta-analyses. Yet, the low coherence of a phenotype is expected to remain low with increasing gene discovery if, in contrast, the absence of coherence is due to the relative importance of non-genetic causes. Our results highlight the low coherence of the molecular mechanisms of intelligence-related traits and cardiovascular and psychiatric disorders, but our method alone cannot decide whether insufficient knowledge or the weakness of the genetic component ultimately explains the lack of internal connectivity characteristic of low-coherence traits.

Our approach is similar in spirit to the well-researched community detection problem [31, 44, 48, 53]. However, we approach this problem from a different angle by asking whether a given network as a whole can be considered a community within the global PPI network. This may be considered a drawback as it has been shown that many networks consist of miniature communities known as motifs [54], or graphlets [55]. While the investigation of motif enrichment [54] is a viable approach to characterize a network, our goal was to develop a unified measure of coherence that can be compared across phenotype-specific networks.

Our method relies on the ability to link the phenotype-associated genetic variants to genes thought to be affected by them. Typically, genes are linked to genetic variants by proximity (nearest gene), a strategy adopted by the NHGRI-EBI GWAS catalog [36]. However, > 88% of phenotype-associated genetic variants are located in regulatory elements outside of protein-coding regions [35]. The pilot project by ENCODE showed that fewer than 10% of all interactions between promoters and regulatory regions involved the closest promoter by linear distance [56], questioning the validity of nearest gene mapping strategy. The development of chromatin conformation capture technologies now allows for understanding long-distance interactions among genomic regions on a genome-wide scale [57, 58], helping to prioritize disease-associated gene-variant associations [59]. Consequently, tools are being developed to consider long-distance interactions when mapping SNPs to genes (3DSNP [60], FUMA [61], PINES [62], HUGIn [63]), and studies redefining disease-associated genes using long-distance interactions started to emerge [64]. Our future goal includes considering long-distance variant-gene interactions in defining phenotype-associated gene sets and comparing their network properties.

The definition of coherence as the slope between internal versus external degree distributions makes our measure dependent on the choice of PPI data [65]. Our limited knowledge of protein–protein interactions limits the coherence estimation to phenotypes that have a sufficient number of genes with PPI annotations, especially for phenotypes with low numbers of associated genes. Furthermore, networks formed by phenotype-associated genes should be sufficiently diverse to avoid cases in which all genes in a network have the same degree. As the different PPI databases vary significantly in the number of PPI annotations, some phenotype-associated gene sets have sufficient PPI annotations only when using one but not the other database. This problem is best illustrated by the BioGrid PPI database that has considerably fewer PPIs; hence, fewer phenotypes could be analyzed. Although our results show good correspondence between coherence estimates when using different PPI databases, care should be exercised when selecting the PPI database for network analyses [65].

The definition of high and random coherence also depends on the choice of PPI data. Therefore, we derived degree distributions for KEGG and random networks separately for each PPI database and implemented the max–min normalization of the coherence estimates. Intuitively, the max–min normalization converts the absolute measures of coherence (slopes of regression lines fit through the internal versus external degree distribution plots) that are dependent on the choice of PPI databases to the percentages of the full range of coherence (from high coherence defined by KEGG networks to low coherence defined by random networks) defined using a given PPI database. The phenotype-specific coherences expressed as a percentage of the full range of coherence can be directly compared across phenotypes and databases.

It should be noted that, although our measure of coherence does not depend on network size, the size of the disease- or phenotype-associated gene networks still affects coherence estimates. Smaller networks may have insufficient interactions to form robust degree distribution. Our study required networks to have at least 10 interactions; however, a larger threshold may be considered. With the increasing number of genomic variants and the corresponding genes identified by GWA studies, our method will yield more precise coherence estimates.

In addition to internal versus external degree distributions, other gene-centric network metrics can be used to estimate coherence [48]. For example, the centrality measure has been used to demonstrate that highly central hub genes are functionally essential and highly conserved [66]. A large study of PPIs (BioPlex) compared centrality distributions among several disease categories. Cancers and immunological disease networks tended to have high centrality, while nervous system, congenital, neonatal, and hereditary disease networks had low centrality [67]. Metabolic pathways were shown to have more duplicated copies of highly central genes making the networks tolerant to loss-of-function mutations [66]. These observations of centrality measure parallel our estimations of network coherence. Our future work will incorporate centrality and other gene-centric network metrics into our framework to further refine our ability to compare the coherence of phenotype-associated molecular mechanisms.

## Conclusion

Our study investigated the coherence of the molecular mechanisms of genes associated with genomic variants of 116 phenotypes. We developed a network coherence measure driven by the relationship between internal and external connectivity (degree distribution) that is robust to the difference in network sizes. Using well-curated lists of genomic variants and the associated genes, we built disease-specific networks using protein–protein interaction information from the STRING and BioGrid databases. We found a range of coherence estimates in each phenotype category, with metabolic phenotypes being the most coherent. Psychiatric disorders had low coherence, with schizophrenia and major depressive disorder being among the least coherent. In summary, we provide a general-purpose method for quantifying and comparing the coherence of molecular networks.

## Methods

### Data sources

Phenotype-associated gene lists were obtained from the NHGRI-EBI catalog of genome-wide association studies (GWAS catalog, gwascat v.2.14.0 R package) [35, 36]. “MAPPED_GENE” column was used to extract trait-associated genes. To organize traits into categories, Experimental Factor Ontology (EFO) IDs were extracted from the “MAPPED_TRAIT_URI” column. Categories were mapped to EFO IDs using the ontoCAT v.1.12.0 R package and the EFO database (accessed 12/04/2018). In the case of a trait being mapped into two categories, the most representative category was manually assigned. Non-canonical gene names were converted to common gene symbols using alias2Symbol function from the limma R package. Gene names which could not be mapped to gene symbols were excluded from the analysis.

Human protein–protein interactions (PPIs) were obtained from STRING database v.11.0 [33] and BioGrid database v.3.5.174 [34]. The STRING data for the tax ID “9606” (human) was used, and Ensembl protein IDs were converted to gene names using BioMart. Either the complete dataset (11,761,040 entries) or data filtered by “combined_score” > 500 (range 0–1000) (1,373,946 entries, the main PPI dataset) was used. The BioGrid data was filtered to include “TaxID interactor” type equal to “9606”, all “Interaction Types” were included, totaling 465,660 interactions. These three PPI databases were used in parallel to evaluate disease coherence.

Phenotype-associated gene networks were formed by mapping trait-associated genes to PPIs. Genes without PPI annotations (e.g., C5orf4, LOC100507462), antisense transcripts (e.g., CDKN2B-AS), readthrough (e.g., NPHP3-ACAD11), non-protein-coding transcripts (e.g., LINC00478) were excluded. Genes that do not have PPIs with each other but interact with other genes in the global PPI network were kept for external degree calculation. Non-Zero interacting genes were defined as genes having at least one interaction with other phenotype-specific genes. Consequently, phenotypes with less than 10 non-zero interacting genes were omitted. Additionally, phenotypes with genes having identical internal degrees (hence, no internal degree distribution) were filtered due to the inability to derive a fit through the internal versus external degree distributions (see below). These precautions were set to avoid situations where a small number of genes in a network can cause instability in the results.

### Comparing internal versus external degree distributions as a measure of coherence

Phenotype-specific internal degree distribution was defined as the number of interactions between phenotype-associated genes, or, more precisely, their interacting protein products. Phenotype-specific external degree distribution was defined as the number of interactions between genes in a phenotype-associated network and all other genes in the PPI network. The internal versus external degree distributions were plotted on a scatterplot. The square root of the degree was taken for more informative representation. For each phenotype, the internal versus external degree distribution plot was fit with a regression line through the origin to capture the slopes. Slopes were used as a measure of coherence. Lower slopes being associated with higher coherence, and vice versa. Degree distributions of the network nodes were obtained using the igraph R package v. 1.0.1.

### Normalization of coherence

To alleviate the dependency of degree distributions from the size of phenotype-associated gene sets and the content of a selected PPI database, we normalized slopes of phenotype-specific networks to the range of slopes formed by degree distributions of highly coherent and random networks. Specifically, we select 30 MSigDb networks (10 from KEGG, GOCC, and REACTOME collections, ${\beta }_{min}$ median slope) and 1000 random networks (${\beta }_{max}$ median slope) matched in size to the corresponding network of phenotype-specific genes. For each phenotype $i$ with slope ${\beta }_{i}$, we calculate the normalized slope ${\beta }_{i \,norm}=\frac{{\beta }_{i}-{\beta }_{max}}{{\beta }_{min}-{\beta }_{max}}$, which gives a measure of coherence on the range of [0,1]. Higher values indicate a higher level of coherence similar to that of MSigDb networks. These values represent normalized coherence and can be compared across phenotypes and PPI databases. In addition, for each coefficient ${\beta }_{i}$, we extract confidence intervals and normalize them as the coefficients themselves. Note the normalization step alters common interpretation of confidence intervals; therefore, results of the permutation test of slopes should be used to compare coherences.

Although using median slopes of MSigDb and random networks is expected to be a robust reference for high/random coherence, some phenotype-specific networks may have coherence larger than 1, that is, being more coherent than the median coherence of MSigDb networks. These situations may occur in smaller networks that have a higher chance to contain genes with unusually high internal degree distributions. Such outliers would inflate coherence estimations. Similar outliers may be observed in larger networks. For these reasons, coherence estimates for excessively large networks (“Obesity-related traits”, 779 genes, “Height”, 418 genes) were excluded from calculating the correlation between coherences obtained using different PPI databases (Additional file 4).

### Permutation test of slopes

To test whether each slope is significantly different than random, we used a permutation test. For phenotype $i$ with the gene network of size ${g}_{i}$ that contains ${n}_{i}$ number of non-zero interactions, we define ${\beta }_{i}$ as the slope derived by regressing the external degree distribution on the internal degree distribution. We then generate networks of randomly selected genes of size ${g}_{i}$ to ensure size-matched topology. Each random network will have a relatively few number of non-zero interactions; therefore, we collect genes non-zero interacting genes from the size-matched random networks until we have $\ge {n}_{i}$ of them, and combine them into a single random network. This procedure is repeated until we have $n={10{,}000}$ random networks of size ${g}_{i}$ having ${n}_{i}$ non-zero interactions. For each random network $j$, we identify its slope ${\beta }_{r}$ representing the internal versus external degree distribution regression line of random coherence. We calculate the permutation p-value as ${p}_{i}=\frac{(\sum_{j=1}^{n}I({\beta }_{rj}>{\beta }_{i})+1}{n+1}$ [68]. Intuitively, this approach estimates whether the slope of the phenotype-specific network ${\beta }_{i}$ is consistently larger than those of random networks. All analyses and visualizations were performed in the R/Bioconductor computing environment v.3.4.0 [69].

### Modularity estimates

Network modularity was estimated using the standard method developed by Clauset et al. [49], implemented in the igraph::modularity function.

## Supplementary information

**Additional file 1. Summary statistics of the analyzed phenotype categories.** Minimum, mean, median, maximum of coherence estimates, the number of genes, SNPs, the total number of phenotype networks. Each worksheet corresponds to the PPI database used.

** Additional file 2. Summary of networks from MSigDB categories.** Minimum, mean, median, maximum of the slopes, the number of genes, and the total number of networks are shown for each collection, along with the correlation between network size and slope. “CP:/KEGG/Reactome/Biocarta”—canonical pathways, “GOBP/GOMF/GOCC”—gene ontology biological processes, molecular functions, cellular component, “TFT”—transcription factor targets, “CGN”—cancer gene neighborhoods, “CM”—cancer modules, “MIR”—microRNA targets, “CGP”—chemical and genetic perturbations.

**Additional file 3: Phenotype-specific coherence estimates.** The PPI database (STRING, STRING filtered, Biogrid) are specified in the corresponding column names. For each phenotype category, the results are sorted alphabetically. In addition to the normalized coherence and the permutation p-values indicating significant difference of phenotype-specific coherence from random, modularity, untransformed slopes, and the corresponding lower and upper confidence interval bounds are shown. “NA” indicates the corresponding value cannot be estimated due to lack of sufficient number of genes annotated with PPIs.

**Additional file 4: Normalization of coherence estimates alleviates its network size dependence.** Red/blue gradient and numbers represent Pearson correlation coefficients for each pairwise comparison of the number of SNPs, genes, normalized coherence estimates, and the untransformed slopes and the sizes of their confidence intervals (CI) using the corresponding PPI databases.

** Additional file 5: Schematic overview of the coherence estimation method.** To estimate the coherence of phenotype-associated genes, a database of protein–protein interactions (e.g., STRING) is used to derive the associated network. In parallel, gene sets known to highly interact (e.g., KEGG canonical pathways) and random gene sets are used to derive size-matched references of high- and low coherence, respectively. The internal versus internal degree distribution plot enables estimating phenotype-associated network coherence with respect to the range of high- and low coherences (normalized coherence). A permutation analysis may be performed to assess whether coherence of the phenotype-associated network is significantly different from that of randomly sampled networks of the same size.

**Additional file 6: Coherence estimates of traits using Biogrid as a reference PPI database.** Size of dots represents the level of normalized coherence (X-axis) for individual traits (Y-axis). Plots are faceted by categories. Missing entries indicate that, for a given trait, a network could not be built and the coherence cannot be estimated.

**Additional file 7: Coherence estimates of traits using STRING as a reference of PPI database.** See legend for Additional file 6.

**Additional file 8: Coherence estimates of diseases using Biogrid as a reference PPI database.** See legend for Additional file 6.

**Additional file 9: Coherence estimates of diseases using STRING as a reference PPI database.** See legend for Additional file 6.

**Additional file 10: Bonferroni-corrected Wilcoxon p values comparing the internal and external degree distributions of phenotype-specific networks having high (> 0.85) and low (< 0.58) normalized coherence.** Each sheet corresponds to the type of degree distribution (internal and external), and the type of PPI database (STRING and STRINGfilt).

## Data Availability

The code and the data supporting the conclusions of this article are available in the https://github.com/dozmorovlab/disease_coherence GitHub repository.

## Abbreviations

GWAS: Genome-Wide Association Study
KEGG: Kyoto Encyclopedia of Genes and Genomes
MSigDb: Molecular Signatures Database
PPI: Protein–protein interaction
SNP: Single nucleotide polymorphism
