# Supplementary figures and images for: A method for estimating coherence of molecular mechanisms in major human disease and traits
Source: BMC Bioinformatics. 2020 Oct 21;21:473. doi: 10.1186/s12859-020-03821-x (PMC7579960; doi:10.1186/s12859-020-03821-x)

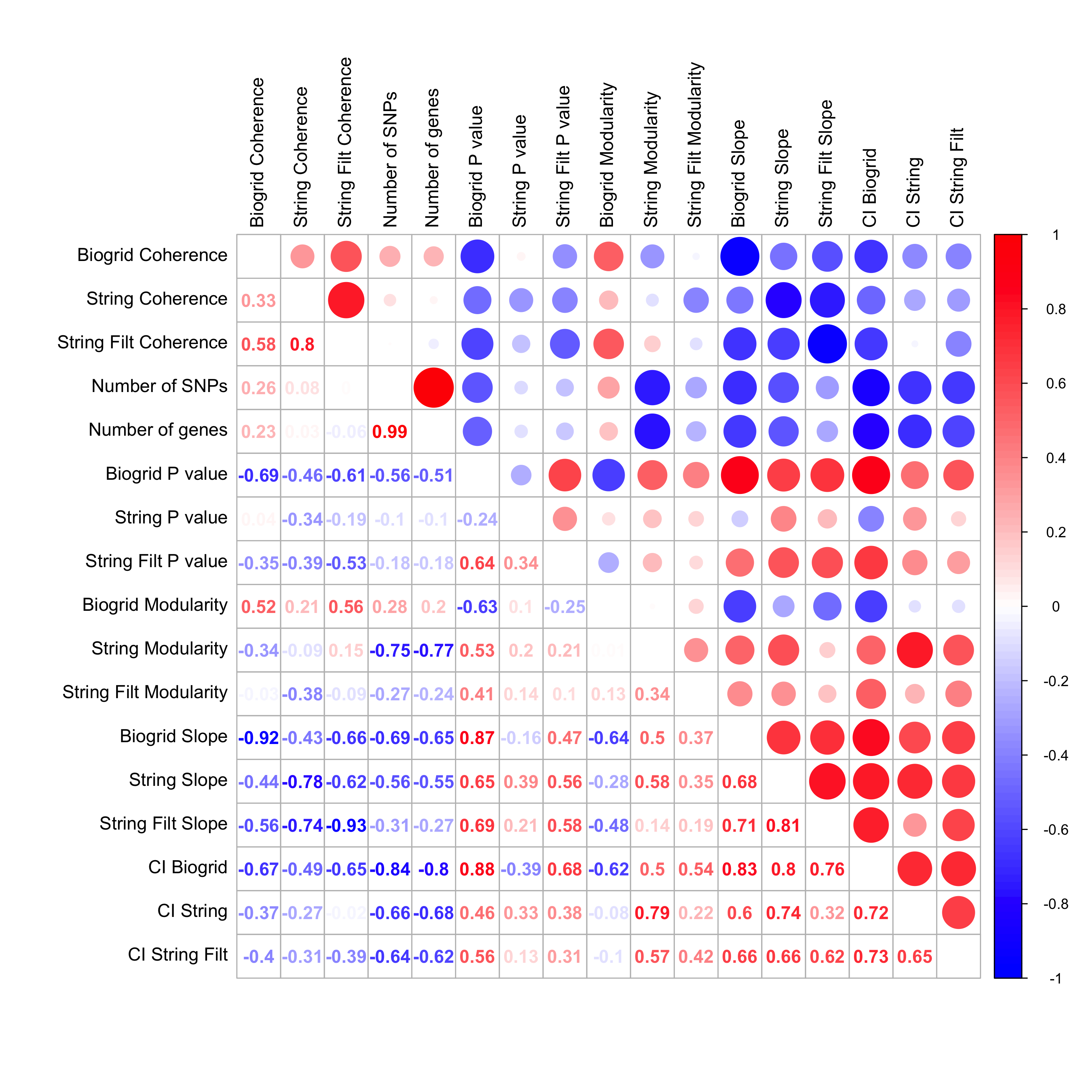

Supplement: Supplementary file 4 — Additional file 4: Normalization of coherence estimates alleviates its network size dependence. Red/blue gradient and numbers represent Pearson correlation coefficients for each pairwise comparison of the number of SNPs, genes, normalized coherence estimates, and the untransformed slopes and the sizes of their confidence intervals (CI) using the corresponding PPI databases. [file 12859_2020_3821_MOESM4_ESM.png]

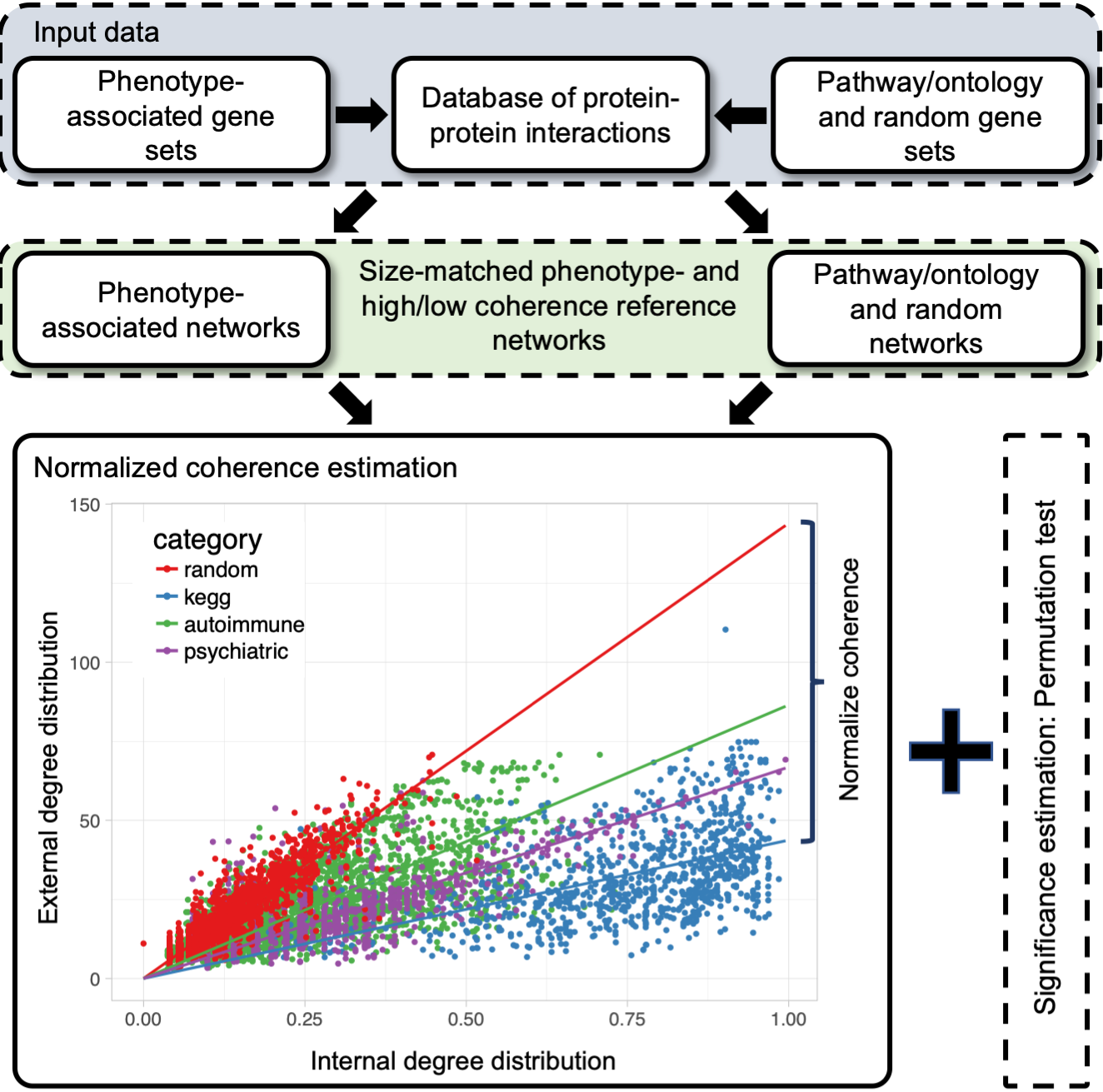

Supplement: Supplementary file 5 — Additional file 5: Schematic overview of the coherence estimation method. To estimate the coherence of phenotype-associated genes, a database of protein–protein interactions (e.g., STRING) is used to derive the associated network. In parallel, gene sets known to highly interact (e.g., KEGG canonical pathways) and random gene sets are used to derive size-matched references of high- and low coherence, respectively. The internal versus internal degree distribution plot enables estimating phenotype-associated network coherence with respect to the range of high- and low coherences (normalized coherence). A permutation analysis may be performed to assess whether coherence of the phenotype-associated network is significantly different from that of randomly sampled networks of the same size. [file 12859_2020_3821_MOESM5_ESM.png]

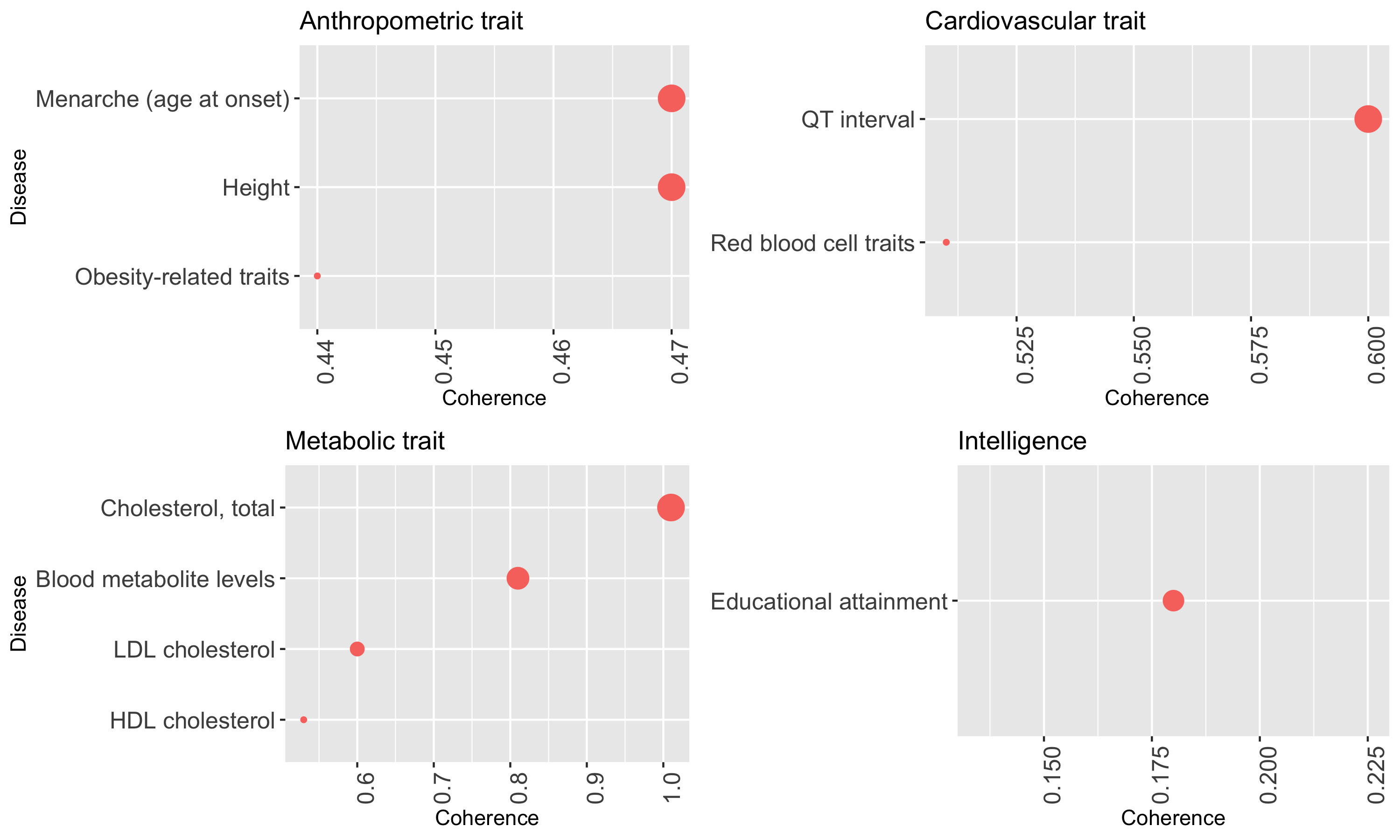

Supplement: Supplementary file 6 — Additional file 6: Coherence estimates of traits using Biogrid as a reference PPI database. Size of dots represents the level of normalized coherence (X-axis) for individual traits (Y-axis). Plots are faceted by categories. Missing entries indicate that, for a given trait, a network could not be built and the coherence cannot be estimated. [file 12859_2020_3821_MOESM6_ESM.png]

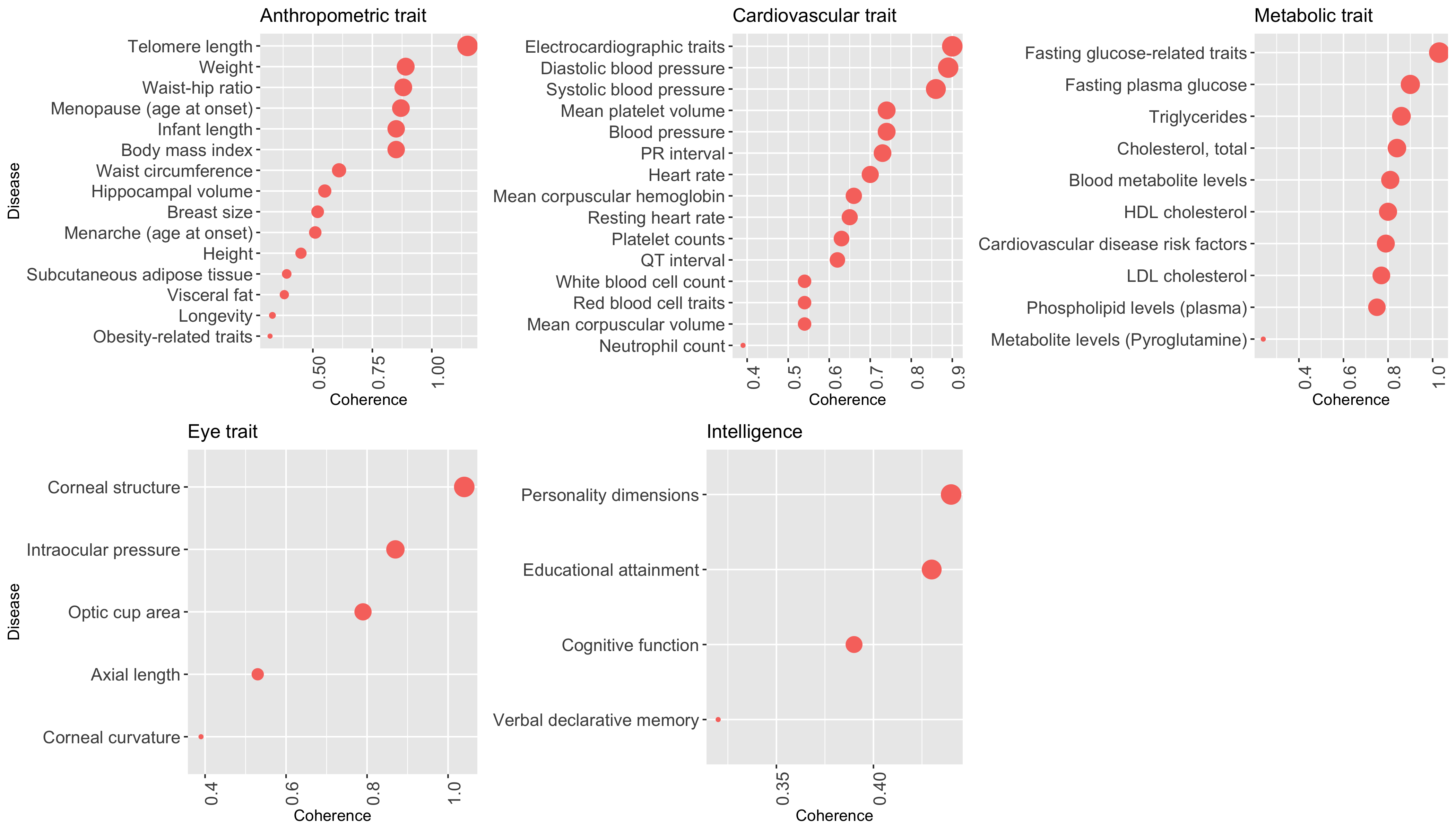

Supplement: Supplementary file 7 — Additional file 7: Coherence estimates of traits using STRING as a reference of PPI database. See legend for Additional file 6. [file 12859_2020_3821_MOESM7_ESM.png]

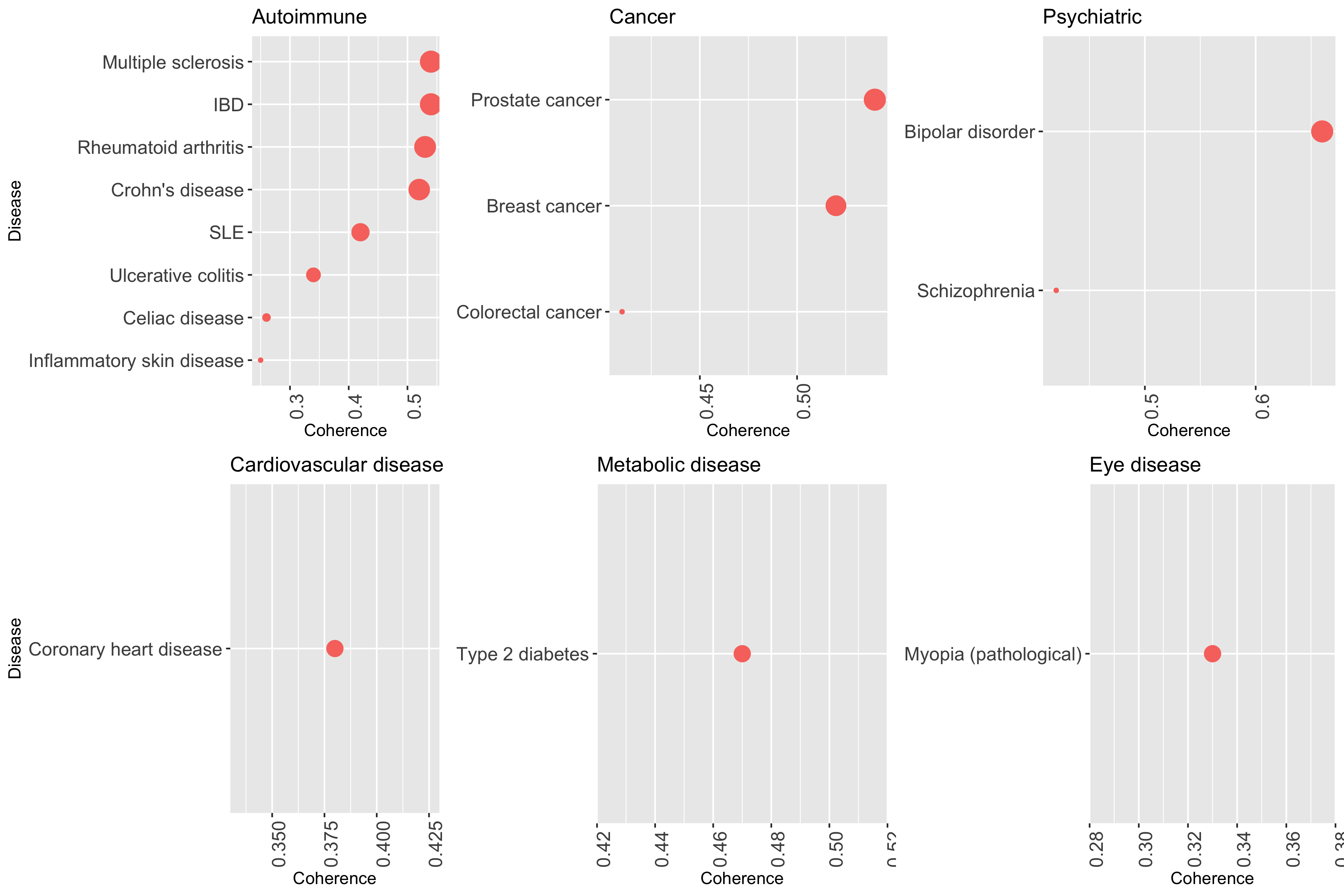

Supplement: Supplementary file 8 — Additional file 8: Coherence estimates of diseases using Biogrid as a reference PPI database. See legend for Additional file 6. [file 12859_2020_3821_MOESM8_ESM.png]

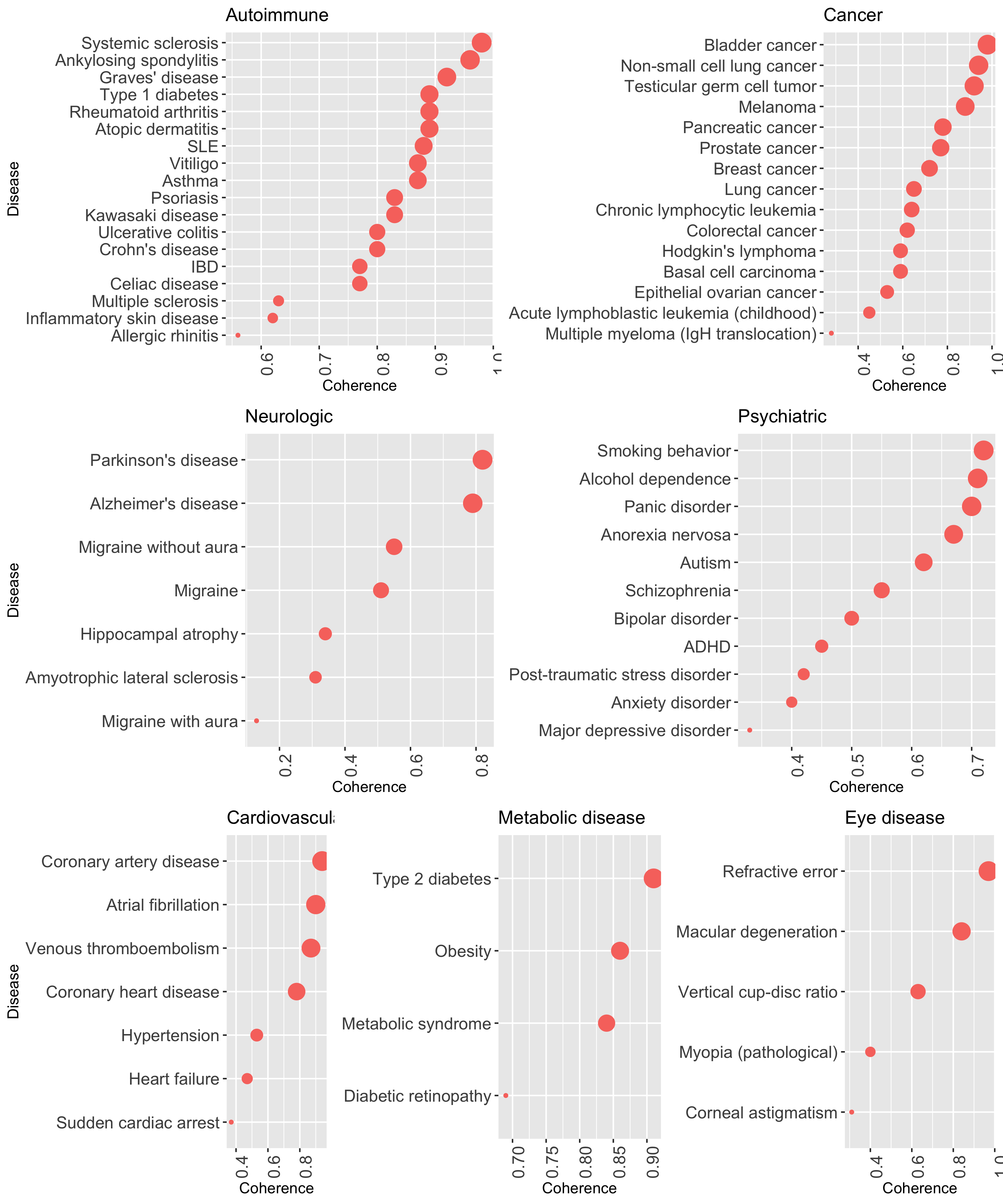

Supplement: Supplementary file 9 — Additional file 9: Coherence estimates of diseases using STRING as a reference PPI database. See legend for Additional file 6. [file 12859_2020_3821_MOESM9_ESM.png]
